# Multifocal Motor Neuropathy With Cranial Nerve Involvement and Vocal Cord Paralysis: A Case Report

**DOI:** 10.7759/cureus.25179

**Published:** 2022-05-21

**Authors:** Maria Clarissa Nunez, Belinda Lioba M Nepomuceno, Ma Luisa Gwenn P Tiongson

**Affiliations:** 1 Department of Clinical Neurosciences, University of the East Ramon Magsaysay Memorial Medical Center, Quezon City, PHL; 2 Department of Neuroscience, University of the East Ramon Magsaysay Memorial Medical Center, Quezon City, PHL

**Keywords:** peripheral neuropathy, cranial nerve involvement, vocal cord paralysis, demyelinating neuropathy, multifocal motor neuropathy

## Abstract

Multifocal motor neuropathy (MMN) is a progressive, multifocal weakness, which typically begins and predominates in the upper extremities with the absence of a sensory deficit and a hallmark electrophysiologic finding of conduction block. We describe a case of an adult male with MMN who developed both cranial nerve involvement and vocal cord paralysis. The patient presented with left shoulder weakness without sensory loss followed by hoarseness of voice and later developed tongue deviation and wasting of the left sternocleidomastoid and left trapezius muscle. Laryngeal electromyography (EMG) showed findings evident for a focal mononeuropathy involving the left recurrent laryngeal nerve. EMG and nerve conduction studies (EMG NCV) of the upper extremities showed evidence for a multifocal mainly motor neuropathy involving the left spinal accessory and hypoglossal nerves, combined with the presence of median and ulnar proximal conduction blocks bilaterally. Given the clinical presentation and electrophysiologic findings of conduction block, the patient was managed as a case of MMN and received the standard treatment with Intravenous Immunoglobulin (IVIg). Upon follow-up, there was an improvement in symptoms and no recurrence of motor weakness and hoarseness of voice. There are a few case reports about MMN but none with multiple lower cranial nerve involvement.

## Introduction

Multifocal motor neuropathy (MMN) is a rare disorder with an incidence of 1:100,000 [1]. It is an immune-mediated demyelinating neuropathy that presents with asymmetric distal limb weakness without sensory loss. Affectation of cranial nerves is a rare presentation, frequently involving the hypoglossal nerve. It can also manifest as vocal cord paralysis during its natural course. It is a disease that can mimic other motor neuron diseases such as amyotrophic lateral sclerosis (ALS), chronic inflammatory demyelinating polyneuropathy (CIDP), multifocal acquired demyelinating sensory and motor (MADSAM), and Lewis Sumner Syndrome (LSS). A diagnostic criterion for MMN has been proposed to help distinguish it from other immune neuropathies. It is important to differentiate MMN from other neuropathy because it is highly responsive to treatment with IVIg. While a few published data have been available regarding MMN presenting with cranial nerve involvement, none presented with multiple lower cranial nerve palsies. Hence, we present an unusual case of a patient with multiple lower cranial nerve involvement with upper limb motor weakness and conduction block.

## Case presentation

The patient is an adult male who experienced left shoulder weakness described as difficulty lifting with his left upper extremity five weeks prior to admission. The patient was managed as a case of muscle spasms secondary to fatigue and was prescribed with muscle relaxant and underwent physical therapy. Four weeks prior to admission, the patient developed hoarseness of voice which was managed as a case of epiglottitis and was prescribed with oral methylprednisone. A video laryngoscopy and modified functional endoscopic evaluation of swallowing were done which showed paresis of the left true vocal fold. Magnetic resonance imaging (MRI) of the neck and chest with contrast showed the failure of abduction of the left true vocal cord, consistent with the clinically known true vocal cord paralysis. No mass or abnormal area of contrast enhancement was visualized, and an MRI of the chest was unremarkable. Laryngeal EMG showed the left thyroarytenoid with denervation changes with increased insertional activity, sustained runs of fibrillation potentials noted at rest, and severely reduced recruitment with only 1-3 units firing at rapid rates. The findings show evidence for a focal mononeuropathy involving the left recurrent laryngeal nerve which is severe but incomplete with the muscle tested receiving its innervation from this nerve showing severely reduced but at least some voluntary activation.

One week prior to admission, there was no improvement in symptoms after intake of oral steroids. The hoarseness of voice persisted, and the patient developed tongue deviation. Upon admission, there were pertinent findings of a weak gag reflex on left, uvula was deviated, and a weak palatal elevation left. There was a decreased muscle tone on the left trapezius with wasting of the left sternocleidomastoid muscle and deviation of the tongue to the left. Muscle strength was 5/5 on all extremities with intact sensory, vibration, and proprioception sense. He was areflexic on the bilateral biceps, triceps, and brachioradialis. The patient is hypertensive and non-diabetic with no history of vasculitis.

Cranial MRI and MRA with contrast were done which showed no evidence of acute infarct or posterior fossa mass. Cerebrospinal fluid analysis results showed a slightly elevated protein at 0.63 g/L with a reference value of 0.15-0.45 g/L, normal CSF sugar, and the serum were negative for antiganglioside IgG and IgM. EMG NCV of bilateral extremities is abnormal showing evidence for a multifocal mainly motor neuropathy involving the left spinal accessory and hypoglossal nerves. The median and ulnar nerves (Table 1) showed normal compound muscle action potential (CMAP) amplitudes, distal motor latencies and conduction velocities, additional proximal stimulation of both median and ulnar nerves at the Erb’s point showed complete conduction block. Stimulation of the spinal accessory nerve (Table 1) showed normal CMAP amplitude on the right with no demonstrable response obtained on the left on repeated trials. Needle EMG study of the left genioglossus and left trapezius muscles showed normal motor unit action potentials (MUAPs) with severely reduced recruitment pattern and florid runs of fibrillation potentials.

**Table 1:** Motor nerve conduction studies Motor conduction studies performed on the median and ulnar nerves showed normal CMAP amplitudes, distal motor latencies and conduction velocities. However, additional proximal stimulation of both median and ulnar nerves at the Erb’s point showed complete conduction block. Stimulation of the spinal accessory nerve showed normal CMAP amplitude on the right. No demonstrable response was obtained in the left spinal accessory nerve on repeated trials.

Nerve and Site	Latency	Amplitude	Segment	Latency Difference	Distance	Conduction Velocity
Left Median nerve						
Wrist	3.3 ms	10.0 mV	Wrist - Elbow	4.1 ms	232 mm	57 m/s
Elbow	7.4 ms	9.4 mV	Elbow-Erbs	No response	No response	No response
Erbs	No response	No response				
Left Ulnar nerve						
Wrist	2.6 ms	6.1 mV	Wrist- Elbow	3.4 ms	245 mm	72 m/s
Elbow	6.0 ms	5.5 mV	Elbow – Above elbow	1.7 ms	110 mm	65 m/s
Above elbow	7.7 ms	5.3 mV	Above elbow - Erbs	No response	No response	No response
Erbs	No response	No response				
Left Spinal Accessory nerve						
Neck	No response	No response	Trapezius - Neck	No response	No response	No response
Right Median nerve						
Wrist	3.5 ms	9.2 mV	Wrist -elbow	3.9 ms	220 mm	56 m/s
Elbow	7.4 ms	9.0 mV	Elbow-Erbs	No response	No response	No response
Erbs	No response	No response				
Right Ulnar nerve						
Wrist	2.8 ms	5.5 mV	Wrist-elbow	3.9 ms	250 mm	64 m/s
Elbow	6.7 ms	5.2 mV	Elbow – above elbow	1.8 ms	100 mm	56 m/s
Above elbow	8.5 ms	4.5 mV	Above elbow - Erbs	No response	No response	No response
Erbs	No response	No response				
Right Spinal Accessory nerve						
Neck	2.8 ms	1.6 mV	Trapezius- neck	2.8 ms	170 mm	m/s

With the clinical presentation of asymmetric weakness without sensory abnormality, associated with cranial nerve involvement, vocal cord paralysis, and unresponsive to steroids plus the EMG NCV findings of conduction block, the patient was managed as a case of MMN. For treatment, intravenous immunoglobulin at 2 g/kg over five days was administered and was discharged thereafter. A few months from discharge, the patient noted improvement in strength and muscle tone of the left upper extremity, deep tendon reflexes without recurrence of hoarseness of voice and weakness.

## Discussion

Cranial nerve involvement and vocal cord paralysis are the rare presentations of MMN. The diagnosis of MMN is made based on clinical and electrodiagnostic features [2]. The electrophysiological hallmark is conduction block outside the usual sites of nerve compression [1,3]. As stated in the guidelines of the joint task force of the European Federation of Neurological Societies (EFNS) and the Peripheral Nerve Society (PNS) the core clinical criteria for MMN, are slowly progressive or stepwise progressive, focal, asymmetric limb weakness, that is, motor involvement in the motor nerve distribution of at least two nerves, for more than one month and no objective sensory abnormalities except for minor vibration sense abnormalities in the lower limbs [3]. Supportive criteria include predominant upper limb involvement, decreased or absent tendon reflexes in the affected limb, absence of cranial nerve involvement, cramps and fasciculations in the affected limb, and response in terms of disability or muscle strength to immunomodulatory treatment [3]. Our patient presented with a five-week history of left upper shoulder weakness without the sensory deficit and on examination with noted decreased muscle tone on the left trapezius with a muscle wasting of the left sternocleidomastoid muscle and areflexia of the bilateral biceps, triceps, and brachioradialis on examination.

While there are published reports about MMN, an extensive search of the literature shows very limited publication about MMN presenting as vocal cord paralysis or cranial nerve involvement. This case report is an unusual case of MMN due to the presentation of both cranial nerve involvement and vocal cord paresis. Our case presented initially with asymmetric weakness of the upper extremity without sensory loss, this was followed by hoarseness of voice caused by the vocal cord paralysis and then eventually with cranial nerve involvement such as weak gag reflex on left, leftward deviated uvula, and a weak left palatal elevation, muscle wasting of the left sternocleidomastoid and left trapezius and tongue deviation to the left. There are a few case reports about MMN with cranial nerve involvement. A case reported by Axelsson and Liedholm presented right-sided hypoglossal palsy with fasciculations and atrophy of the hands [4]. Kaji et al. reported two patients with MMN with conduction block who had atrophy of the tongue and limb muscles [5]. Turker et al. presented a case with progressive weakness of hand muscles associated with weakness of orbicularis oculi muscles and fasciculations of the tongue [6]. A patient with ophthalmoplegia and subsequently multiple cranial nerve palsies in association with bibrachial paresis was presented by Pringle et al. [7]. Vocal cord paralysis in MMN has been reported in the past. A case by Olchovsky et al. reported a patient who developed MMN with vocal cord paralysis following Borrelia infection [8]. Another case report was done by De La Blanchardiere et al. on a patient with AIDS and CMV infection who presented with severe motor weakness and paralyzed left true vocal cord [9]. A case report of vocal cord paralysis with MMN in an immunocompetent patient was presented by Balaban et al. [10]. Among the literature about MMN with cranial nerve involvement our case is unique because it presented with multiple lower cranial nerve palsies.

The electrophysiologic criteria as defined by the joint task force of the EFNS and the PNS, conduction block is defined as a negative peak CMAP area reduction on proximal vs. distal stimulation of at least 50% whatever the nerve segment length (median, ulnar, and peroneal) or a negative peak CMAP amplitude on stimulation of the distal part of the segment with motor CB must be >20% of the lower limit of normal and >1 mV and increase of proximal to distal negative peak CMAP duration must be ≤30% [3].

The laryngeal EMG of our patient showed mononeuropathy involving the left recurrent laryngeal nerve while EMG of bilateral upper extremity showed evidence for a multifocal mainly motor neuropathy involving the left spinal accessory and hypoglossal nerves and combined with the presence of bilateral median and ulnar proximal conduction blocks. Although the exact mechanism is not completely clear, in theory, it results from primary dysfunction of the axon at the nodes of Ranvier or paranodal abnormality of the myelin sheath [11]. Among patients with MMN, 20%-80% are found to have anti-GM1 antibodies; however, it is not specific to MMN and a negative finding does not exclude the disease [12]. Intravenous immunoglobulin is the standard of treatment. In contrast to the response in chronic inflammatory demyelinating polyradiculoneuropathy, MMN is not responsive to steroids [3]. As seen in our patient who was initially given oral steroids, the symptoms persisted and progressed. Some studies state that there is a high frequency of axonal degeneration in MMN and that axon loss, rather than conduction block is the most important determinant of permanent weakness and disability [11]. Therefore, intravenous immunoglobulin treatment may lessen axonal dysfunction and promote reinnervation thereby neutralizing the pathogenic pathway that results in axonal loss [11]. The usual starting dose is 2 g/kg body weight given on 2-5 consecutive days [1]. The frequency of IVIg maintenance therapy should be guided by the response. The typical treatment regimens are 1 g/kg every 2-4 weeks or 2 g/kg every 1-2 months [2]. There was the resolution of the hoarseness of voice, left upper extremity weakness, improvement of muscle tone, and tendon reflexes a few months after discharge.

## Conclusions

MMN is a disease that is often misdiagnosed or undiagnosed because it shares common features with other immune-mediated neuropathies. Compared to other neuropathies it is treatable, therefore it is essential to be cognizant of this illness in order to avoid misdiagnosis and administer proper treatment immediately. Early diagnosis is crucial given that early intervention with IVIg may improve its prognosis.
